# Unravelling Chemical Composition of Agave Spines: News from *Agave fourcroydes* Lem.

**DOI:** 10.3390/plants9121642

**Published:** 2020-11-25

**Authors:** Dalia C. Morán-Velázquez, Juan L. Monribot-Villanueva, Matthieu Bourdon, John Z. Tang, Itzel López-Rosas, Luis F. Maceda-López, José L. Villalpando-Aguilar, Lorena Rodríguez-López, Adrien Gauthier, Laura Trejo, Parastoo Azadi, Francisco Vilaplana, José A. Guerrero-Analco, Fulgencio Alatorre-Cobos

**Affiliations:** 1 Colegio de Postgraduados Campus Campeche, Carretera Haltunchén-Edzná km 17.5, Sihochac, Campeche 24450, Mexico; moran.dalia@colpos.mx (D.C.M.-V.); maceda.luis@outlook.com (L.F.M.-L.); lodoyarena96@gmail.com (L.R.-L.); 2 Red de Estudios Moleculares Avanzados (REMAV), Instituto de Ecología A. C. Carretera Antigua a Coatepec 351, Xalapa 91070, Mexico; juan.monribot@inecol.mx; 3 Sainsbury Laboratory, University of Cambridge, Cambridge CB2 1LR, UK; matthieu.bourdon@slcu.cam.ac.uk; 4 Complex Carbohydrate Research Center, University of Georgia, Athens, GA 30602, USA; johnzt@uga.edu (J.Z.T.); azadi@ccrc.uga.edu (P.A.); 5 CONACYT-Research Fellow Colegio de Postgraduados Campus Campeche, Carretera Haltunchén-Edzná km 17.5, Sihochac, Campeche 24450, Mexico; itzel.rosas@colpos.mx; 6 Tecnológico Nacional de México, Instituto Tecnológico de Chiná, Calle 11entre 22 y 28, China 24050, Mexico; jose.va@china.tecnm.mx; 7 UniLaSalle—AGHYLE Research Unit UP 2018.C101, 3 Rue du Tronquet—CS 40118-76134, 76134 Mont-Saint-Aignan, France; adrien.gauthier@unilasalle.fr; 8 CONACYT-Research Fellow Laboratorio de Biodiversidad y Cultivo de Tejidos Vegetales, Instituto de Biología, UNAM, Santa Cruz, Tlaxcala 90640, Mexico; laura.trejo@st.ib.unam.mx; 9 Division of Glycoscience, Department of Chemistry, School of Engineering Sciences in Chemistry, Biotechnology and Health, KTH Royal Institute of Technology, SE-100 44 Stockholm, Sweden; franvila@kth.se

**Keywords:** fibers, cellulose, lignin, secondary metabolites, tannins, *Agave fourcroydes*

## Abstract

Spines are key plant modifications developed to deal against herbivores; however, its physical structure and chemical composition have been little explored in plant species. Here, we took advantage of high-throughput chromatography to characterize chemical composition of *Agave fourcroydes* Lem. spines, a species traditionally used for fiber extraction. Analyses of structural carbohydrate showed that spines have lower cellulose content than leaf fibers (52 and 72%, respectively) but contain more than 2-fold the hemicellulose and 1.5-fold pectin. Xylose and galacturonic acid were enriched in spines compared to fibers. The total lignin content in spines was 1.5-fold higher than those found in fibers, with elevated levels of syringyl (S) and guaiacyl (G) subunits but similar S/G ratios within tissues. Metabolomic profiling based on accurate mass spectrometry revealed the presence of phenolic compounds including quercetin, kaempferol, (+)-catechin, and (−)-epicatechin in *A. fourcroydes* spines, which were also detected in situ in spines tissues and could be implicated in the color of these plants’ structures. Abundance of (+)-catechins could also explain proanthocyanidins found in spines. Agave spines may become a plant model to obtain more insights about cellulose and lignin interactions and condensed tannin deposition, which is valuable knowledge for the bioenergy industry and development of naturally dyed fibers, respectively.

## 1. Introduction

Thorns, spines, and prickles are modifications that plants have developed as sessile organisms to protect themselves from herbivores [1,2]. Generally, thorns are derived from shoots or branches, spines from leaves, and prickles from epidermal tissue [1,3]. Defining the ontogeny of these structures is not an easy task without detailed anatomical studies, therefore thorn and spine are words often used indistinctly or just named as spine equivalent [4,5]. Recently, for the first time, a genetic framework underlying thorn development was reported in citrange, where the binding of teosinte branched1/cycloidea/proliferating cell factor 1 (TCP) transcription factors to WUSHEL (WUS) regulates negatively the stem cell activity in thorn meristems [2]. The genetic modulators controlling spines and prickles differentiation are unknown so far.

With ca. 200 species described, agaves are endemic species of arid and semiarid regions of Central and Northern Mexico and the Southwestern United States but now dispersed throughout the world [6,7]. Agave leaves, with unique shapes and arrangement, are decorated with a terminal sharp pointed structure, referred to as a spine, and hardened teeth along the leaf edge. Of 194 species described, 57.7% have apical spines and 44.3% teeth along of leaf margins [4,8]. Apical spines can be brown reddish, gray, black, white, or yellow, rigid or flexible, straight or curved, and prominently long in some species, especially in those belonging Salmianae section [4,8,9,10]. In contrast to Cactaceae spines, for which chemical composition and ultra-structural characteristics have been described [11,12,13,14], structural composition and anatomy of agave spines remain uncovered. All described agave species possess highly colored spines that suggest an interesting regulatory interplay between metabolite accumulation and sclerenchyma development, and they may become an outstanding model to design fibers that produce its own color. Moreover, agave spines show a species-specific variability of tensile properties that may be related to structural component ratios. Terminal spine is a morphological character commonly used to describe agave species [15,16,17,18,19]. However spine metabolomic profiling remains uncovered still, it could represent a useful tool in chemotaxonomy. In the Asparales order, which Agavaceae belong to, saponin glycosides, mostly from roots, have showed to be good markers for chemotaxonomic classifications of Asparagus genotypes [20,21,22]. In xerophyte species of Cactaceae, omics tools have revealed a good taxonomic correspondence between lignin derivatives from spines and the species [14]. Here, hyphenated chromatographic and histochemistry approaches were used to characterize spines chemical composition of *Agave fourcroydes* Lem., a species traditionally used for fiber extraction, and more particularly their structural carbohydrates, lignin monolignol subunits profiling, and metabolite landscape of terminal spines. Based on the results, the possible roles of carbohydrates and polyphenols in the structural properties and coloring of agave spines are discussed, respectively. Potential use of some metabolites found in spines as markers for chemotaxonomy and the suitability of these structures as models to study naturally colored fibers are also considered. 

## 2. Results and Discussion

### 2.1. Lignocellulosic Composition of Fiber and Spines

**Figure 1:**
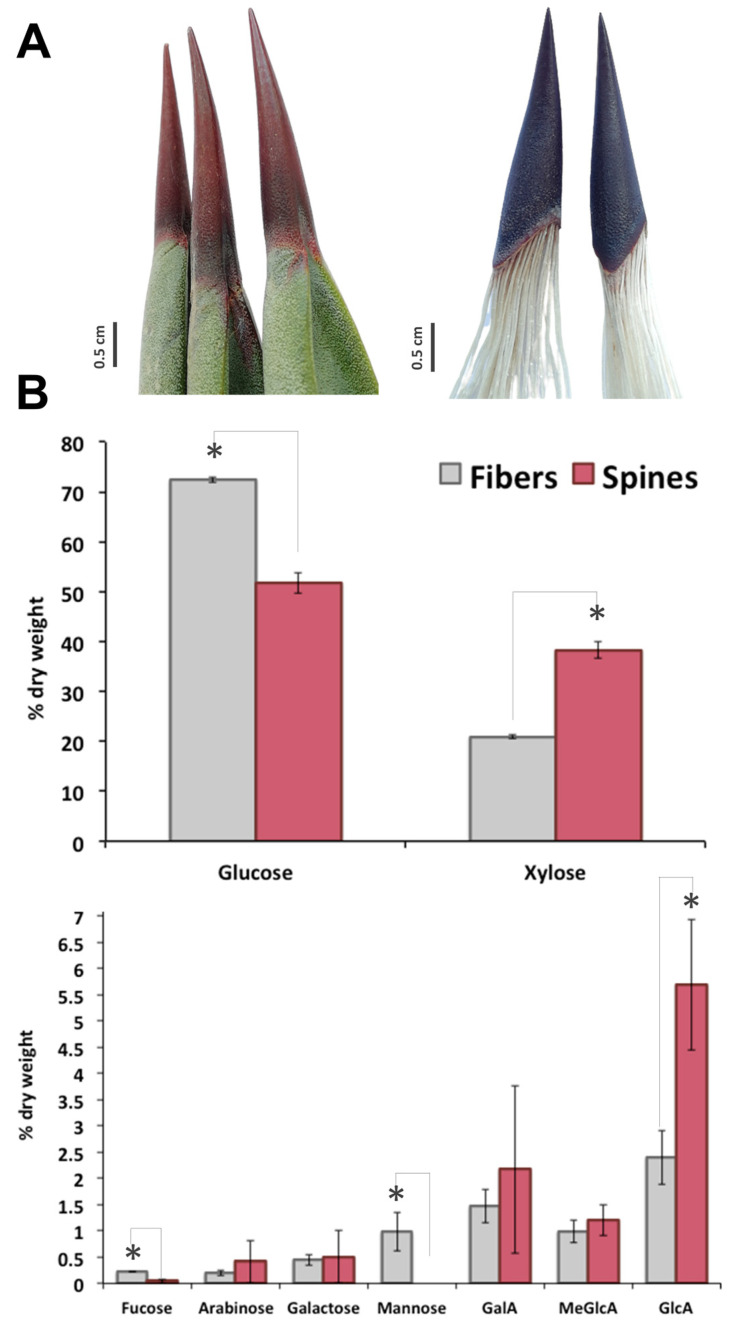
Sugar composition of terminal spines and fibers of *A. fourcroydes* leaves. (**A**) Morphology of collected terminal spines (left) and after process (right) to show fibers and spines tissues connection. (**B**) Monosaccharides analysis by high performance anion exchange chromatography with pulsed amperometric detection (HPAEC-PAD). Values are means ± SD. Asterisks indicate significant statistical differences between fibers and spines determined by Tukey’s honest significant difference (HSD) test (*p* ≤ 0.05). GalA: galacturonic acid; MeGlcA: 4-O-methyl-D-glucuronic acid; GlcA: glucuronic acid.

In *A. fourcroydes* leaf, although terminal apical spines are tightly connected to fiber sclerenchyma (Figure 1A), structural carbohydrate composition of both tissues is completely different. Sugar analysis revealed 72% cellulose (as Glc content), 24% hemicellulose (mainly attributed to xylan), and 2% pectic polysaccharides (as galacturonans) in fibers and 52% cellulose, 45% hemicellulose, and 3% pectin in spines, respectively (Figure 1B). In line with this, xylose—the backbone moiety of xylan, the main component content for hemicellulose fraction [23,24,25]—was 2-fold higher in spines than in fibers (Figure 1B). Quantification of side branches for xylan, glucuronic acid (GlcA) and 4-O-methyl-D-glucuronic acid (meGlcA) and arabinose indicated that both substituents were 2–2.5 fold higher in spines compared to fibers (Figure 1B). Another two residues for xyloglucans, fucose and galactose, were also compared between fibers and spines in this study. Fucose concentration was 5-fold higher in fibers than in spines, while no significant differences for galactose were found in both tissues. Mannose was at the order of 1% of dry weight of fibers but not detected in agave spine (Figure 1B). Analysis of galacturonic acid (GalA), major structural element for pectin, showed an enhanced accumulation in agave spines (Figure 1B). Overall, carbohydrate profiling observed in *A. fourcroydes* spines is contrasting with previously reported in *Opuntia ficus-indica*. Indeed, Opuntia cladode’s spines consist mainly of cellulose and arabinose in equal proportions, without any other polysaccharide components [12]. In agave spines, arabinose levels were found significantly lower than glucose residues, which suggest tensile properties completely different from that those found in Opuntia spines [11]. Spines elasticity may be affected by high contents of GalA, which is associated with the biogenesis and constitution of pectin. Pectin is a heteropolysaccharide associated to arabinans, galactans, and galacturonans and found in the middle lamella of primary and secondary cell walls, which affects cell wall porosity and rigidity [26,27]. Pectin content in terminal spines may explain the qualitative variation reported for spine elasticity in agave species [10] that could be determined evaluating GalA and GlcA as two chemical markers. 

**Figure 2:**
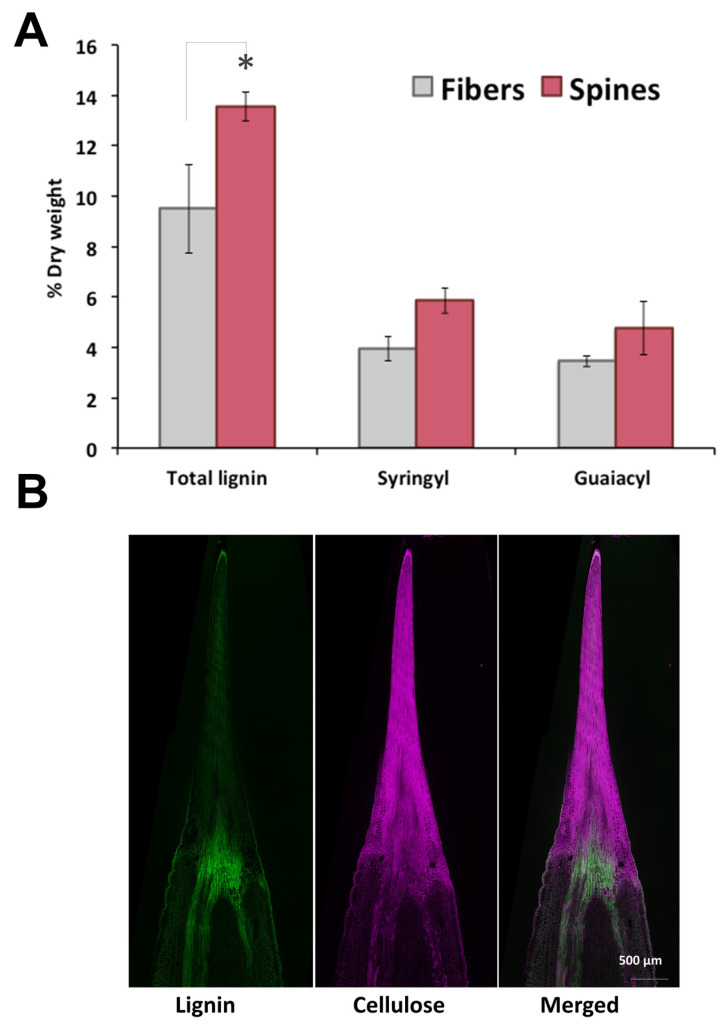
Lignin and metabolic profiling of terminal spines of *A. fourcroydes* leaves. (**A**) Total lignin and (syringyl and guaiacyl) monolignol subunit contents in fiber and spines collected from fully developed leaves, quantified by pyrolysis gas chromatography/mass spectrometry (py-MBMS). Values are means ± SD. Asterisks indicate significant statistical differences between fibers and spines determined by Tukey’s HSD test (*p* ≤ 0.05). (**B**) In situ detection of lignin and cellulose in longitudinal sections of young spines collected in 2-year-old plants. Lignin was detected by autofluorescence and cellulose with Direct Red 23 staining under confocal microscopy.

To further analyze cell wall structural components of agave fibers and spines, total lignin and monolignols composition were measured. Total lignin content in spines was 1.5-fold higher than in fibers, with elevated levels of syringyl (S) and guaiacyl (G) monomeric subunits but similar S/G ratios within tissues (Figure 2A). In contrast to cellulose, lignin autofluorescence revealed a uniform distribution throughout spines and fibers, except for at tissues junction (Figure 2B). Total lignin content and S/G ratio found here for *A. fourcroydes* were within the reported range for other agave species such as *A. tequilana* and *A. americana* [28,29,30] contrary to *A. sisalana* fibers, enriched in acetylated S-lignin units [31,32,33]. Knowledge about lignin and monolignols composition in agave spines is poorly understood, and to the best of our knowledge, this is the first report about structural components of such tissues. 

### 2.2. Metabolomic Profiling and In Situ Secondary Polyphenols Detection of Spine Tissues

**Figure 3:**
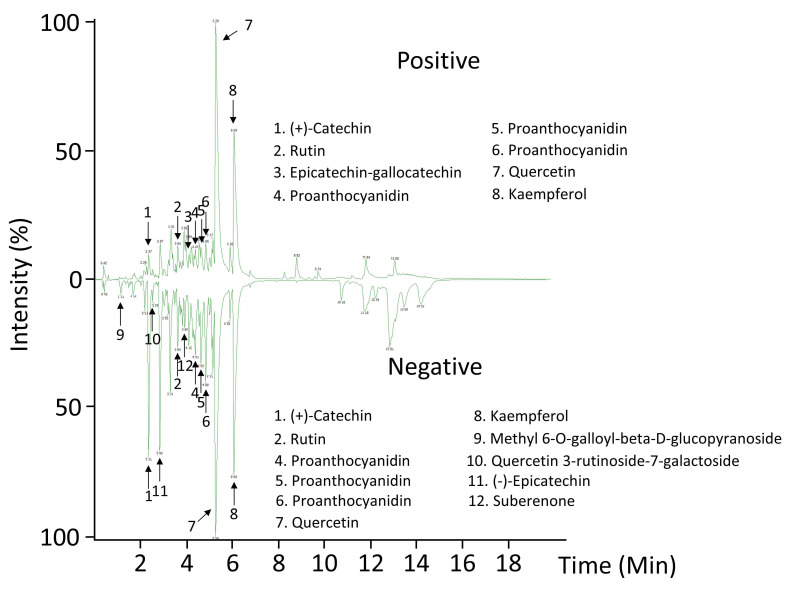
Representative chromatograms of terminal spines revealed by LC-MS-QTOF ESI+/−. The peaks for the most abundant compounds identified in negative (–) and positive (+) electrospray (ESI) ionization modes are indicated by numbers.

Recently, plant spines have been reported to be particularly enriched with phenylpropanoid-derived compounds, specifically from lignocellulosic matrix [14,34,35]. In this work, an ultra-performance liquid chromatography coupled to mass spectrometry has been developed for the analysis of secondary metabolites in *A. fourcroydes* spines, which could be involved in aposematic coloration, a feature typically encountered in agave spines [4,8]. Abundance patterns allowed us to tentatively identify flavonoids (quercetin, kaempferol) and condensed tannins ((+)-catechin and (−)-epicatechin) as the predominant metabolites in spines (Figure 3 and Table 1). These compounds may contribute to the *A. fourcroydes* spine color. Although quercetin and kaempferol are well established to be found ubiquitously in plants [36,37], this work reported their presence for the first time in agave spines at our knowledge. Flavonoids, homoisoflavonoids, and phenolic acids have been widely reported in agave species, especially in leaves [38,39,40]. Here, flavonoids and condensed tannins found in spines may count to total contents quantified previously in whole leaves. Moreover, our study suggests (+)-catechin and (−)-epicatechin as two abundant precursor molecules in spines, which is in line with proanthocyanidins (PAs) found in such an organ (Figure 3 and Table 1). 

**Figure 4:**
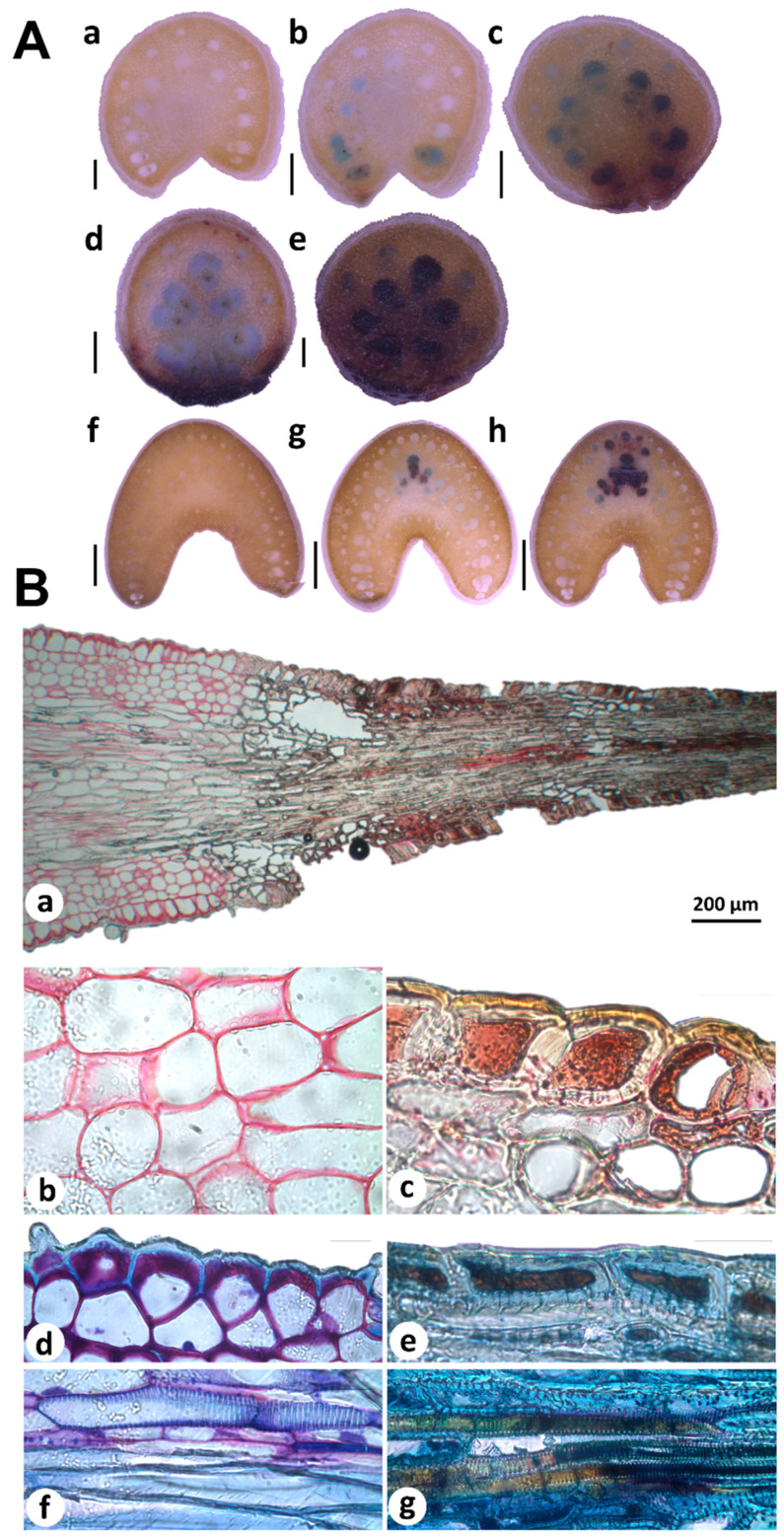
Histochemical analysis of condensed tannins in agave spines tissues. (**A**) In situ detection of proanthocyanidins (PAs) by using 4-(Dimethylamino)-cinnamaldehyde (DMACA) in free-hand cross sections of young (**a**–**e**) and mature (**f**–**h**) spines, collected in 2-year-old plants and 8–10-year-old plants, respectively. PAs are revealed as blue spot in neighboring cells (blue-stained cells) of differentiated cells into spines (brown-stained cells). Scale bars = 0.5 mm (**a**–**e**), 2 mm (**f**–**h**). (**B**) Condensed tannins staining in young spines by using ruthenium red (RR) and toluidine blue O (TBO). Condensed tannins are visualized throughout the spine and adjacent tissues (**a**). Zoom amplifications show pectins colored in pink in parenchyma cell walls (**b**), while condensed tannins were detected as brown-red cells (**c**). Double staining with RR and TBO revealed carboxylated polysaccharides in lilac-colored cell walls of epidermis (**d**), tannins in cell walls (brown-yellow) and cytosol (brown) of spine cells (**e**), lignin in fibers (blue; (**f**)), and tannins deposited in spines fibers (brown; (**g**)). Scale bars in (**b**–**g**) = 20 μm.

Histochemical analyses of free-hand sections of young and mature terminal spines revealed PAs accumulation patterns associated to spine development (Figure 4A). Spine cross sectioning from bottom to top showed PAs accumulation in neighboring cells (blue-stained cells) of differentiated cells (brown-stained cells; Figure 4A(a–e)). In mature spines, few cells showed PAs accumulation; blue-stained cells were found more frequently in spine-bottom tissues (Figure 4A(g)) contrary to full differentiated areas (Figure 4A(f)). Cross sections obtained from areas far from spine tissues (Figure 4A(a,f)) presented no staining confirming the robustness of PAs analysis. All these data suggest that PAs are closely associated to spine differentiation. In situ detection of secondary compounds in histological sections of young spines also supports this idea. Staining of cells adjacent (parenchyma) and spine tissues showed different staining patterns revealed by ruthenium red (RR) and toluidine blue O (TBO). Parenchymatous cell walls appeared in pink after RR staining, indicated pectin substances (Figure 4B(a,b)), as previously reported for primary cell walls [41]. In contrast, spine tissues showed brown-yellow cell walls and brown-red intracellular content (Figure 4B(a,c)), confirming spines tissues accumulate condensed tannins. Double-staining with RR and TBO showed pectin and carboxylated polysaccharides in lilac stained cell walls of parenchyma and epidermis cells (Figure 4B(d,f)) but not in spine tissues. Metachromatic TBO was very informative to distinguish fiber cells than accumulate lignin and tannins. Fiber cells located out of spine tissues accumulated lignin but no tannins according TBO staining (Figure 4B(f)). Fibers incrusted in spines were stained in brown (Figure 4B(g)), indicating secondary compounds presence, most likely such as tannins [41,42].

PA biosynthesis by catechin oxidation can be mediated by laccases as previously revealed by in vitro assays, gene expression profiling, and mutant analysis [43,44,45,46]. Interestingly, occurrence of PAs and tannin-containing fibers in *A. fourcroydes* spines suggests laccase-catalyzed fiber coloration. In spines fiber, laccases may be working into lignin subunits polymerization but also assuming a functional role during PAs biosynthesis. This idea perfectly fits with the moonlighting nature of these proteins [47,48] and arises a scenario with potential agave spine laccases in future biotechnological applications in the textile industry.

## 3. Materials and Methods

### 3.1. Leaves and Spines Sampling and Preparation

**Figure 5:**
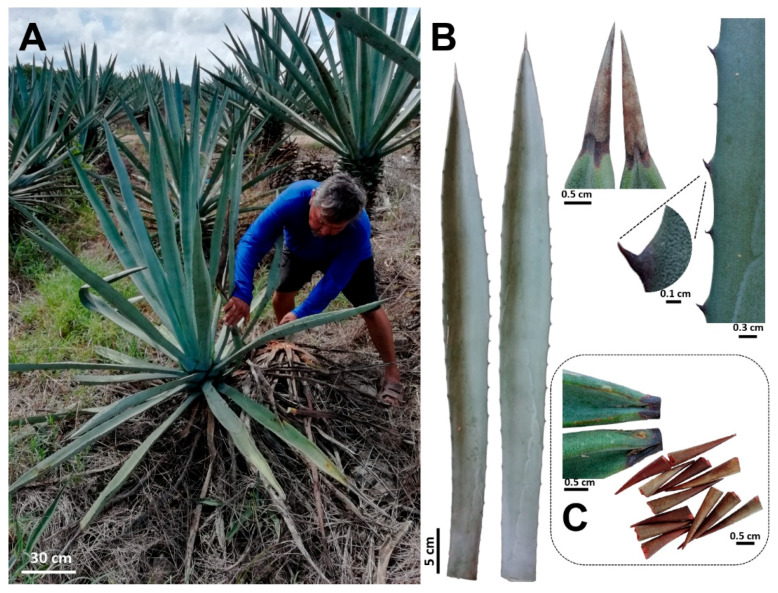
*Agave fourcroydes* plants harvested for fibers and spines analyses. (**A**) Adult plants showing the typical rosette growth pattern attributed to *A. fourcroydes*. (**B**) Leaf morphology (leaf shape, terminal, and lateral spines). (**C**) Terminal spines harvested for chemical composition analysis.

Fully expanded leaves of 8–10-year-old plants of *A. fourcroydes* were collected in March 2018, in the Hacienda Santa Teresa farm, Yucatán, México (21°13′53.8′′ N 89°15′22.5′′ W). Previous studies have also used *A. fourcroydes* plants from the same farm [49]. For this study, the typical leaf features and plant growth habit previously reported [19] were observed in all harvested plants (Figure 5A,B). Fibers were mechanically extracted at the farm, washed with distilled water, and then dried at 65 °C for 3 days. Terminal spines were obtained from the collected leaves using a pruner scissor. To avoid leaf parenchyma contamination, spines were cut 2–3 mm after the border between the green tissue and spine (Figure 5C), then washed briefly with distilled water and dried at 65 °C for 3 days. To get fibers attached to spines, leaves were double autoclaved (121 °C, 20 min, 15 psi), parenchyma tissue was removed manually, and fiber-spine structures were washed with tap water and air-dried.

### 3.2. Monosaccharide Composition Analysis

Monosaccharide content and composition was determined in triplicate by acid hydrolysis followed by chromatography analysis. First, fibers and spines were freeze-dried overnight. Fibers were subjected to cell wall extraction protocol to avoid non-structural polysaccharide contamination in monosaccharide analysis. Briefly, fiber samples were sequentially treated with chloroform: methanol (2:1) for 1 h, twice with 70% ethanol for 1.5 h, 80% ethanol for 1 h, and 95% ethanol for 2 h. All these steps were subjected to stirring at room temperature. Samples were then briefly washed with acetone and dried under a stream of air. Prepared cell walls were starch-free using pancreatic α-amylase (16 U/g) (Sigma-Aldrich, Germany). Prepared cell walls from fibers and ground spines were hydrolyzed by Saeman hydrolysis, using a first step with 72% sulfuric acid for 3 h at room temperature, and a second step diluting with deionized at 100 °C and incubated for 3 h. Inositol was added to all samples as an internal standard prior to acid hydrolysis. All filtered hydrolysates were analyzed by high performance anion exchange chromatography with pulsed amperometric detection (HPAEC-PAD) (ICS-3000, Dionex, Thermo Fisher Scientific, Sunnyvale, USA) equipped with a CarboPac PA1 column (4 × 250 mm, Dionex) as previously reported [50]. All chemicals were purchased from VWR (Fontenay Sous Bois, France) or Fisher Scientific (Loughborough, UK), and all monosaccharide standards from Sigma-Aldrich (Steinheim, Germany) or Megazyme (Wicklow, Ireland). 

### 3.3. Lignin Composition

Total lignin and monolignol contents were measured in duplicate by pyrolysis gas chromatography/mass spectrometry (py-MBMS) as previously reported [51]. Ground samples were sieved through a 1 mm screen, weighed, and pyrolyzed (PY-2020 iS, Frontier Labs) for 2 min at 500 °C to produce volatile compounds. NIST8492 (*Populus deltoids*) and aspen (*Populus tremuloides*) analytical standards were included in each run of the experiment for quality control. The volatile compounds were analyzed for lignin using the MBMS (Extrel Max-1000, Super-Sonic). Ultrapure helium compressed gas was used as a carrier gas (0.6–0.7 L/min, pressure 90–100 kPa). The raw data were processed through UnscramberX 10.1 software. Compounds with mass-to-charge ratio values corresponding to major peaks assigned to lignin were processed. Lignin peaks with m/z 120, 124, 137, 138, 150, 152, 154, 164, 167, 178, 180, 181, 182, 194, and 210 were summed and averaged for the samples. Syringyl (S) peaks with m/z 167, 168, 182, 194, 208, and 210 and guaiacol (G) peaks with m/z 124, 137, 138, 150, 164, and 178 were identified. The S/G ratio was obtained by summing the S peaks and dividing the sum by the sum of the G peaks. 

### 3.4. Extraction and Metabolic Profiling of Spines

Methanolic extracts of spines were prepared as previously described [50]. Briefly, 0.6 g of dried and ground material were mixed with 0.3 g of diatomaceous earth to obtain the extracts using an accelerated solvent extraction system (Dionex, ASE 350). The extraction temperature was 60 °C, and it was used one cycle with 5 min of static time. The rinse volume was 30% of used solvent, and nitrogen was the carrier gas. Aliquots were filtered with polytetrafluoroethylene membranes (0.2 µm) and placed in 1.5 mL UPLC vials. Metabolic profiling was performed as reported [52,53]. The analyses were performed in a Class I liquid chromatography coupled to a Synapt G2-Si high-resolution quadrupole time of flight mass spectrometer (LC-MS-QTOF) (Waters, Milford, USA). For the chromatography separation, an Acquity BEH C18 (1.7 μm, 2.1 × 50 mm; Waters) column was used. The temperatures of column oven and samples were 40 and 15 °C, respectively. As mobile phases, water (A; MS grade, Sigma-Aldrich, St. Louis, USA) and acetonitrile were used (B; MS grade, Sigma-Aldrich, St Louis, USA), both with 0.1% of formic acid (MS grade, Sigma-Aldrich, St Louis, USA). The elution gradient started with 1% of B, then in 15 min a linear gradient from 1 to 80% of B, later isocratic at 80% of B for 1 min, and finally, a linear gradient from 80 to 1% of B in 1 min. The flow rate of the mobile phase was 0.3 mL min^−1^ and 5 µL of extract solution was injected. An electrospray source (ESI) in positive and negative modes with capillary, sampling cone, and source offset voltages of 3, 40, and 80 kV, respectively, were used. The source and desolvation temperatures were 100 and 450 °C, respectively. The desolvation gas flow was 600 L h^−1^, and the nebulizer pressure was 6.5 MPa. Leucine–enkephalin was used as the lock mass (556.2771, [M + H]^+^; 554.2615, [M − H]^−^). Data were acquired in MSe mode, and the conditions used for MS analyses were mass range of 50–1200 Da; Function 1 CE of 6 V; Function 2 CER of 10–30 V; scan time of 0.5 sec. To identify tentatively metabolites, the acquired data were processed with Waters Software Masslynx V4.1 using the chromatogram and spectrum viewer tools. The mass spectra were compared with the public spectral databases of Metlin [54], Foodb [55], and Massbank [56], respectively. 

### 3.5. Cellulose and Lignin Visualization in Spines 

JB-4 blocks with agave spines were processed for embedding in plastic resin glycomethacrylate following the manufacturer’s instructions (Electron Microscopy Sciences, Hatfield, USA). Blocks were trimmed on with a Leica EM UC7 ultramicrotome (Leica Microsystems, Wetzlar, Germany) with a glass knife. Whole blocks were then mounted directly on a coverslip with a mounting media containing 0.1% Direct Red 23 (Sigma-Aldrich, St. Louis, USA) and imaged with a Leica SP8-iPhox inverted microscope (Leica Microsystems, Wetzlar, Germany). Lignin was detected by autofluorescence and cellulose with Direct Red 23 (excitation 562 nm; emission excitation 580–615 nm).

### 3.6. Histological Staining for PAs and Condensed Tannins

**Figure 6:**
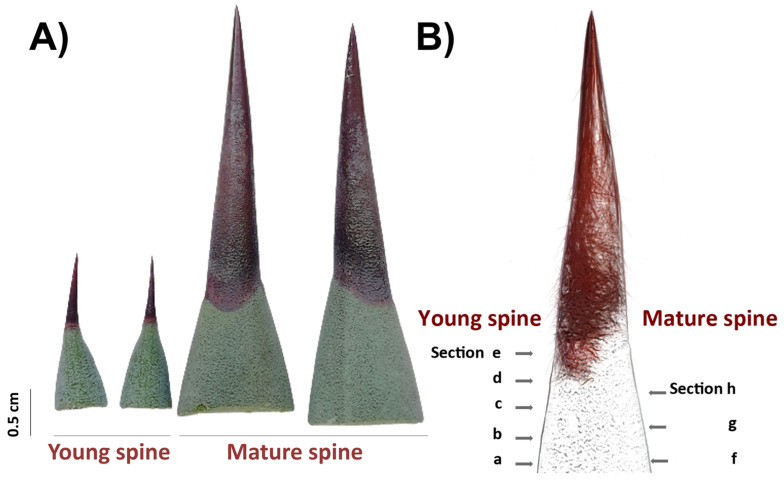
Cross sectioning of adjacent tissues and terminal spines for in situ detection of secondary compounds. (**A**) Free-hand sections were obtained from 2-year-old plants (young spines, left) and 8-year-old plants (mature spines, right). (**B**) Scheme of cross sectioning.

PAs accumulation patterns in spine tissues were analyzed by histological staining as reported [57,58]. A 0.3% 4-(Dimethylamino)-cinnamaldehyde (DMACA) solution was prepared using a stock 1% DMACA solution in distilled water and 1% hydrochloric acid) (Sigma-Aldrich, St. Louis, USA) and a mixture of methanol:6 N HCl (*v*/*v*). Free-hand sections of terminal spines from 2-year-old plants (young spines) and 8-year-old plants (mature spines; Figure 6) were partially discolored with absolute ethanol for 3 h at room temperature, rinsed with sterile distilled water, and incubated with a 0.3% DMACA solution drop for 1.5 h at room temperature. After staining, sections were rinsed with distilled water, mounted on slides, and directly observed by light microscopy in bright fields (Leica M165C, Leica Microsystems, Wetzlar, Germany). For in situ detection of condensed tannins, a protocol previously described [41] based on use of ruthenium red (ammoniated ruthenium oxychloride) and toluidine blue O (TBO) was carried out. Terminal spines of 2-year-old plants were processed for JB-4 resin embedding as described above and sectioned using a Leica RM2255 automatic microtome (Leica Microsystems, Wetzlar, Germany). For staining, 0.05% (*w*/*v*) red ruthenium and 0.1% TBO (*w*/*v*) solutions (Sigma-Aldrich, USA) were prepared with distilled water, filtered with a Millex-GP syringe 0.22 μm filter unit (Merk Millipore, St. Louis, USA), and used to stain 5 μm sections. For ruthenium red, histological sections were incubated for 2 min at room temperature and washed with distilled water. For double staining, sections were firstly incubated with ruthenium red for 1 min, rinsed with distilled water, then stained with TBO for 1 min, and washed again with water. Water-mounted stained sections were imaged using a light microscopy in bright field (Leica DM2000, Leica Microsystems, Wetzlar, Germany). 

### 3.7. Statistical Analyses and Images Processing

All data for monosaccharide and lignin contents were analyzed as completely randomized factorial design. One-way independent ANOVA analyses were carried out test for significant differences among factors analyzed using SAS software (SAS Institute, Cary, USA). If differences were significant, Tukey’s honest significant difference (HSD) tests were performed to find out which treatment’s means (compared with each other) were different (*p* ≤ 0.05). All images were prepared using Remove.bg (Kaleido Al GmbH, Vienna, Austria), Microsoft PowerPoint (Microsoft, Redmond, USA), and GIMP 2.10.12 free software (The GIMP Development Team, Orinda, USA).

## 4. Conclusions

Our study reports for the first time chemical composition (structural lignocellulosic compounds, and metabolomic profiling) of terminal spines of *A. fourcroydes* leaves, compared to fibers. Contrary to fibers, terminal spines are enriched with hemicellulose, pectins, and monolignol subunits, flavonoids, and condensed tannins. Potential differential contents for all of them would explain differences of tensile properties and coloration observed amongst all the agave species described, and within cultivars. Moreover, metabolites found in spines may be used as chemotaxonomic markers and help to solve taxonomy problems remaining in Agavaceae family. Finally, our idea of spine laccase-catalyzed fiber coloration is exciting but further studies about agave spine anatomy, in situ metabolite detection, and laccase activity are needed to support this hypothesis.

## Figures and Tables

**Table 1:** Metabolites putatively identified in *A. fourcroydes* spines. Retention time (RT), mass-to-charge ratio (m/z), quasi-molecular ion, adducts, and fragments are included for each compound, respectively. Numbers indicated in the first column are those shown in the intensity plot of the Figure 3.

Number	RT (min)	m/z	Formula	Ion/Adduct	Error (ppm)	Fragments				Candidate
**Positive mode**										
1	2.37	291.0871	C_15_H_15_O_6_^+^	[M + H]^+^	2	165.055	147.0441	139.0391	123.0439	(+)-Catechin
2	3.64	633.1428	C_27_H_30_O_16_Na^+^	[M + Na]^+^	2	303.0502	257.0445	153.0185	137.0231	Rutin
3	4.13	577.1341	C_30_H_25_O_12_^+^	[M + H − H_2_O]^+^	2	425.0869	317.0659			Epicatechin-gallocatechin
4	4.41	559.1236	C_30_H_23_O_11_^+^	[M + H − H_2_O]^+^	0					Proanthocyanidin
5	4.65	559.1235	C_30_H_23_O_11_^+^	[M + H − H_2_O]^+^	0					Proanthocyanidin
6	4.86	559.1237	C_30_H_23_O_11_^+^	[M + H − H_2_O]^+^	0					Proanthocyanidin
7	5.29	303.0504	C_15_H_11_O_7_^+^	[M + H]^+^	1	257.0444	229.0497	153.018	137.0233	Quercetin
8	6.089	287.0555	C_15_H_11_O_6_^+^	[M + H]^+^	1	241.0499	165.0181	153.0181	121.0282	Kaempferol
**Negative mode**										
9	1.17	345.0829	C_14_H_17_O_10_^−^	[M − H]^−^	0					Methyl 6-O-galloyl-beta-D-glucopyranoside
1	2.37	289.0717	C_15_H_13_O_6_^−^	[M − H]^−^	0	245.0814	203.0707	137.0237		(+)-Catechin
10	2.54	771.2003	C_33_H_39_O_21_^−^	[M − H]^−^	2	609.1455	463.087	301.0339	151.0027	Quercetin 3-rutinoside-7-galactoside
11	2.85	289.0718	C_15_H_13_O_6_^−^	[M − H]^−^	0	245.0813	203.0705	137.0234		(−)-Epicatechin
2	3.64	609.1458	C_27_H_29_O_16_^−^	[M − H]^−^	0	547.1238	331.0454	300.0263	151.0028	Rutin
12	3.95	243.0659	C_14_H_11_O_4_^−^	[M − H]^−^	1	225.0555	201.0552	183.0295	159.0447	Suberenone
4	4.41	557.1097	C_30_H_21_O_11_^−^	[M − H − H_2_O]^−^	2					Proanthocyanidin
5	4.66	557.1092	C_30_H_21_O_11_^−^	[M – H − H_2_O]^−^	1					Proanthocyanidin
6	4.87	557.1084	C_30_H_21_O_11_^−^	[M − H − H_2_O]^−^	0					Proanthocyanidin
7	5.29	301.0351	C_15_H_9_O_7_^−^	[M − H]^−^	0	271.0244	227.0342	151.0031	121.0288	Quercetin
8	6.09	285.0406	C_15_H_9_O_6_^−^	[M − H]^−^	0	255.0298	229.0498	151.0031	93.0339	Kaempferol
